# Gestational hypoxia in late pregnancy differentially programs subcortical brain maturation in male and female rat offspring

**DOI:** 10.1186/s13293-022-00463-x

**Published:** 2022-09-30

**Authors:** E. Nicole Wilson, Steve Mabry, Jessica L. Bradshaw, Jennifer J. Gardner, Nataliya Rybalchenko, Rachel Engelland, Oluwadarasimi Fadeyibi, Oluwatobiloba Osikoya, Spencer C. Cushen, Styliani Goulopoulou, Rebecca L. Cunningham

**Affiliations:** 1 Department of Physiology and Anatomy, University of North Texas Health Science Center, Fort Worth, TX 76107 USA; 2 Texas College of Osteopathic Medicine, University of North Texas Health Science Center, Fort Worth, TX 76107 USA; 3 Department of Pharmaceutical Sciences, UNT System College of Pharmacy, School of Pharmacy, University of North Texas Health Science Center, 3500 Camp Bowie Boulevard, Fort Worth, TX 76107 USA; 4 Present Address: Department of Basic Sciences, Lawrence D. Longo, MD Center for Perinatal Biology, Loma Linda University School of Medicine, Loma Linda, CA 92350 USA

**Keywords:** Prenatal programming, Oxidative stress, Sex differences, Chronic intermittent hypoxia, Ultrasonic vocalizations, Substantia nigra

## Abstract

**Background:**

Hypoxia is associated with pregnancy complications, such as preeclampsia, placental abruption, and gestational sleep apnea. Hypoxic insults during gestation can impact the brain maturation of cortical and subcortical pathways, such as the nigrostriatal pathway. However, the long-term effects of in utero hypoxic stress exposure on brain maturation in offspring are unclear, especially exposure during late gestation. The purpose of this study was to determine the impact of gestational hypoxia in late pregnancy on developmental programming of subcortical brain maturation by focusing on the nigrostriatal pathway.

**Methods:**

Timed pregnant Long–Evans rats were exposed to chronic intermittent hypoxia or room air normoxia from gestational day (GD) 15–19 (term 22–23 days). Male and female offspring were assessed during two critical periods: puberty from postnatal day (PND) 40–45 or young adulthood (PND 60–65). Brain maturation was quantified by examining (1) the structural development of the nigrostriatal pathway via analysis of locomotor behaviors and the substantia nigra dopaminergic neuronal cell bodies and (2) the refinement of the nigrostriatal pathway by quantifying ultrasonic vocalizations (USVs).

**Results:**

The major findings of this study are gestational hypoxia has age- and sex-dependent effects on subcortical brain maturation in offspring by adversely impacting the refinement of the nigrostriatal pathway in the absence of any effects on the structural development of the pathway. During puberty, female offspring were impacted more than male offspring, as evidenced by decreased USV call frequency, chirp USV call duration, and simple call frequency. In contrast, male offspring were impacted more than female offspring during young adulthood, as evidenced by increased latency to first USV, decreased simple USV call intensity, and increased harmonic USV call bandwidth. No effects of gestational hypoxia on the structural development of the nigrostriatal pathway were observed.

**Conclusions:**

These novel findings demonstrate hypoxic insults during pregnancy mediate developmental programming of the cortical and subcortical pathways, in which male offspring exhibit long-term adverse effects compared to female offspring. Impairment of cortical and subcortical pathways maturation, such as the nigrostriatal pathway, may increase risk for neuropsychiatric disorders (e.g., mood disorders, cognitive dysfunction, brain connectivity dysfunction).

**Supplementary Information:**

The online version contains supplementary material available at 10.1186/s13293-022-00463-x.

## Highlights

- Brain maturation of the nigrostriatal pathway is sex- and age- dependent. Exposure to hypoxia in late pregnancy impacts brain maturation of the nigrostriatal pathway that can be observed during puberty and young adulthood.
- Gestational hypoxia impacted female offspring during puberty more than males, whereas it impacted male offspring during young adulthood more than females.
- These novel findings demonstrate that hypoxic insults during pregnancy mediate developmental programming of the cortical and subcortical pathways, in which male offspring exhibit long-term adverse effects compared to female offspring.
- Long-term adverse effects of gestational hypoxia in offspring can occur in the absence of pregnancy complications, especially if they occur within critical embryological developmental periods.

**Supplementary Information:**

The online version contains supplementary material available at 10.1186/s13293-022-00463-x.

## Background

Hypoxia or decreased oxygen during pregnancy is associated with many gestational complications, such as preeclampsia and gestational sleep apnea [1–5]. Gestational sleep apnea is a common late gestational (i.e.,third trimester) hypoxic stressor that is observed in 26% of pregnant women [6, 7]. Gestational hypoxic stress can induce a multitude of long-term impairments across the physical, cognitive, and psychosocial domains, such as long-term memory problems [8–10] and motor impairments [5]. Perinatal insults at late gestation (i.e,. third trimester) are specifically relevant to fetal brain development, because this is the period that cortical and subcortical maturation (e.g., neuroplasticity) occurs [11–16], such as the nigrostriatal pathway [11, 13, 17–19].

Male and female offspring respond differently to insults during gestation [20–22]. Prenatal insult paradigms in rodents showed that male offspring, and not female offspring, exhibited impairments of all maturation domains: psychosocial domain (elevated brain-regulated stress responses during adulthood [23, 24], social behavior [25]), cognitive domain (learning and memory deficits [21, 25]), and physical domain (decreased growth following weaning [23, 24] and impaired motor function [26]). Further, males are more sensitive to common insults that occur anytime during gestation, such as placental inflammation [27, 28], hypoxia [27, 28], term preeclampsia and eclampsia [26]. Although many studies have found that males may be more vulnerable to prenatal insults, recent studies demonstrated that prenatal insults can also negatively impact female offspring [26, 29, 30]. Animal studies that include females are not as extensively conducted, and thus the impact of prenatal insults on female offspring is understudied [31–33].

Ultrasonic vocalizations (USVs) are dependent on multiple brain maturation domains, especially a functional nigrostriatal pathway in the brain [34–36]. USVs are calls emitted by rodents to other rodents to relay information [37–39]. They are innate behaviors and not learned [40, 41]. Fifty kilohertz (kHz) USVs were first described in the early 1970s, and they were associated with rodent mating behaviors [42]. However, 50 kHz USVs are not restricted to mating behaviors [43, 44], as they are emitted under several conditions, such as mother–pup retrieval, juvenile play interactions, same-sex interactions, and opposite-sex interactions [45–48]. Chirp and simple calls are the most common USV call type, especially during cage mate separation, and are generally associated with social coordination functions (i.e., re-establishing social contact) rather than reward or pleasure [41, 43, 49]. The more complex USVs, such as harmonic call types, are associated with sex behaviors, group housing, juvenile play, and other pleasurable behaviors (e.g., tickling) [50–54]. Larger USV bandwidths are associated with communicating to other rodents further away, indicating that the higher bandwidth may be used to compensate in a more expansive environment [55]. In addition to 50 kHz USVs, rodents vocalize at 22 kHz USVs under stressful of aversive conditions [56]. These 22 kHz USVs associated with distress or danger are generally long duration calls (> 300 ms) [56, 57].

The long-term effects of in utero hypoxic stress exposure on brain maturation in offspring are unclear, especially exposure during late gestation. Therefore, the purpose of this study was to determine the impact of late gestational hypoxia on the developmental programming of subcortical brain maturation by focusing on the nigrostriatal pathway. We used an intermittent hypoxia protocol between GD 15–19 to model gestational sleep apnea during the third trimester, which is observed in 26% of pregnant women [6, 7]. We focused on the nigrostriatal subcortical pathway that is established during GD 16–20 [11, 13, 17–19]. Brain maturation was quantified by examining (1) the structural development of the nigrostriatal pathway via analysis of locomotor behaviors and the dopaminergic neuronal cell bodies located in the substantia nigra and (2) the refinement of the nigrostriatal pathway by quantifying USVs that are sensitive to any nigrostriatal pathway impairments [34–36]. In addition to behavioral measures of the nigrostriatal pathway integrity, we examined oxidative stress, as the substantia nigra exhibits high basal levels of reactive oxygen species [58], by quantifying circulating oxidized proteins and oxidative stress-associated enzymatic activity in the substantia nigra. We hypothesized that exposure to hypoxia during late gestation would impair fetal brain maturation in a sex-dependent manner.

## Methods

### Animals

All experiments were conducted in agreement with the Guide for the Care and Use of Laboratory Animals of the National Institutes of Health and the ARRIVE guidelines. These protocols were approved by the Institutional Animal Care and Use Committee of the University of North Texas Health Science Center.

#### Dams

All experiments were conducted using timed pregnant Long-Evans rats (aged 8–10 weeks, Charles River, Wilmington, MA). Dams arrived at the University of North Texas Health Science Center animal facilities on gestational day 5–7 (term = 22–23 days). Animals were single-housed in a 12 h:12 h light/dark cycle with lights on at 0900 h and were provided food and water ad libitum. Two studies were conducted. The purpose of the first study (*n* = 16 dams) was to examine offspring behavior in response to late gestational hypoxia, while the purpose of the second study (*n* = 12 dams) was to characterize the effects of gestational hypoxia on fetal and placental biometrics. Animals were allowed to habituate for 1 week prior to the initiation of the chronic intermittent hypoxia (CIH) protocol.

#### Offspring

**Fig. 1:**
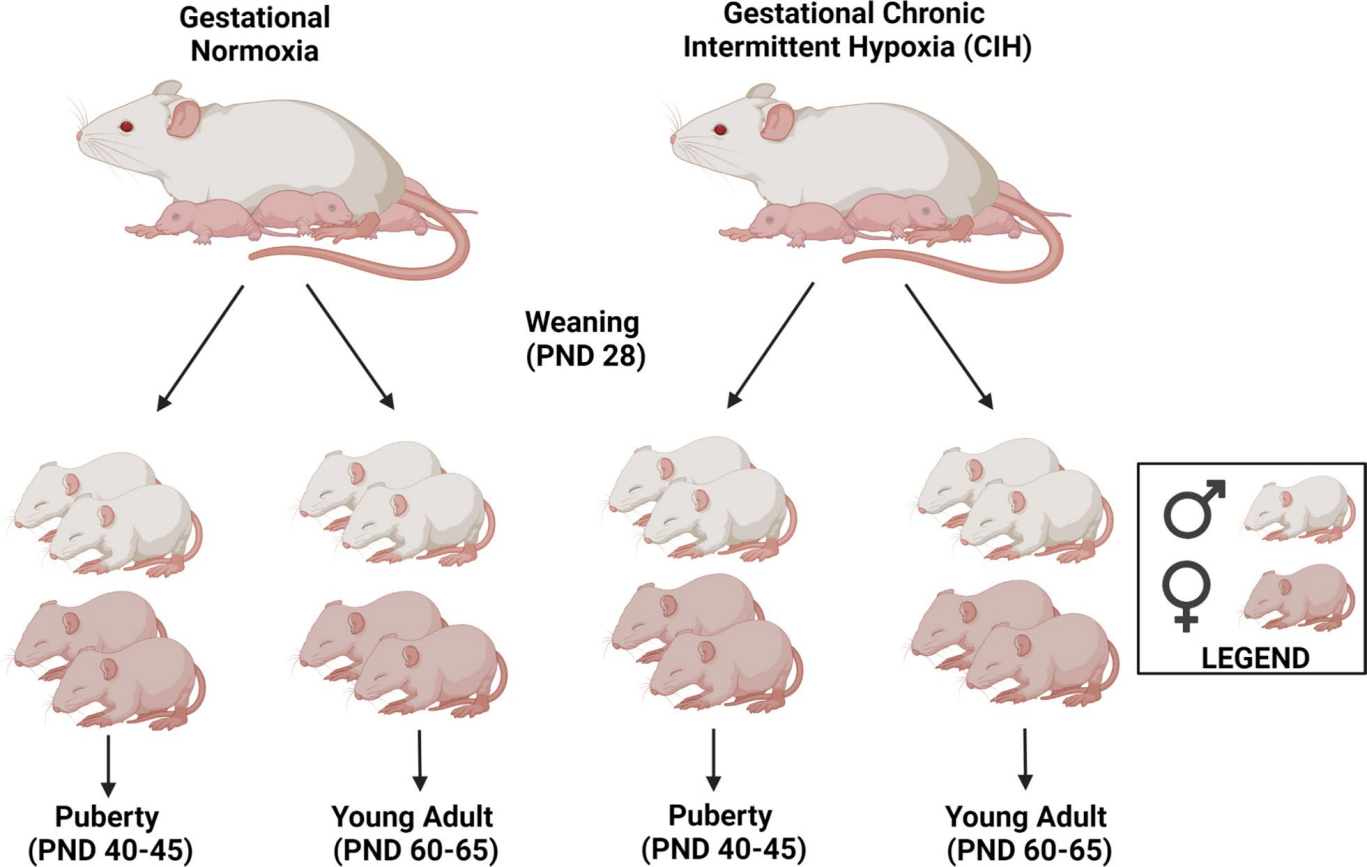
Description of study design. For study one and two, Long–Evans pregnant female rats were exposed to chronic intermittent hypoxia (CIH) or room air (normoxia) on gestational days (GD) 15–19 (*n* = 6-8/group). For study one, dams were allowed to give birth (term = GD 22–23) and litters were reduced to eight pups/litter and, when possible, to equal number of males and females. Offspring were weaned at postnatal day (PND) 28 and separated into two groups: Puberty (*n* = 2/sex/dam; behavior testing = PND 40–45; euthanasia = PND 48) and Young Adult (*n* = 2/sex/dam; behavior testing = PND 60–65; euthanasia = PND 66). For study two, dams and fetuses were euthanized on GD 20

For study one, pup weights and measures of crown-rump length and abdominal girth were collected twelve to sixteen hours after birth. Sex was estimated by determination of anogenital distance, which was confirmed by the presence of gonads (testes or vagina) during the prepubertal stage. Litters were reduced to eight pups/litter and, when possible, to equal number of males and females. Pups were culled based on size, wherein heavier or lighter than the group average of pup offspring was removed. Weaning occurred on postnatal day (PND) 28. Immediately after weaning, dams were anesthetized with 2–3% isoflurane and euthanized via decapitation. Subsequently, four male and four female pups in each litter were randomly assigned to either pubertal (*n* = 2/sex/litter) or young adult (*n* = 2/sex/litter) groups for behavioral studies (Fig. 1). For study two, dams and fetuses were euthanized via decapitation on GD20 to collect fetal and placental biometrics [59].

Pubertal and young adult offspring (sexually naïve) were pair housed with their littermate of the same sex. Male offspring were moved to a separate room from the female offspring for the remainder of the study. Offspring were housed in rooms on a 12-h (h) reverse light cycle where lights were off at 0900 h. Reverse lighting allowed for behavioral testing to be conducted during offspring’s wake phase of the circadian cycle. Food and water were provided to all animals ad libitum*.* Offspring body weights were obtained weekly after weaning. To acclimatize the offspring to operator handling and reduce stress responses during behavior testing, animals were handled daily, beginning approximately 10 days prior to the start of behavior testing. Pubertal and young adult offspring were anesthetized with 2–3% isoflurane and euthanized via decapitation on PND 48 or PND 66, respectively.

### Gestational chronic intermittent hypoxia

To examine the impact of hypoxia at late gestation on offspring behaviors, timed-pregnant female rats were assigned to receive either chronic intermittent hypoxia (CIH) or normoxia (Study one: *n* = 8/group; Study two: *n* = 6/group) starting at 0900 h for 8 h during their sleep phase of the circadian cycle on gestational days 15–19. To induce CIH, the home cages of the pregnant rats were placed into Oxycycler chambers (76.2 × 50.8 × 50.8 cm, BioSpherix, Lacona, NY, USA). Animals were allowed to acclimatize to the chambers under normoxic (room air) conditions for a period of 4 days prior to starting the CIH protocol, in which oxygen was reduced from 21% (room air) to 10% and then returned to 21% using a 6-min cycle (10 cycles/h) over 8 h/day for a period of 5 days, as previously described [60–63]. Based on gestational exposure to hypoxia or normoxia, offspring from these dams were included in one of the following groups (Fig. 1): male normoxic (MN), male CIH (MC), female normoxic (FN), and female CIH (FC).

Study two examined the effects of late gestation CIH on fetal and placental biometrics. This separate study used two different CIH protocols: (1) 6-min cycles (10 cycles/hour) over 8 h/day for a period of 5 days and (2) 8-min cycles (8 cycles/hour), as previously described [60, 63]. This resulted in three different groups: (1) CIH with 6-min cycles/hour (*n* = 3), (2) CIH with 8-min cycles/hour (*n* = 3), and normoxic controls that were simultaneously run with each of the CIH protocols (*n* = 6). No statistical significance was found between the two CIH protocols on any pregnancy outcome measures, and thus these CIH groups were collapsed into one group (*n* = 6).

### Behavioral testing

Behavioral studies were conducted during puberty (PND 40–45) and young adulthood (PND 60–65) over two days during their wake period from 0945 to 1700 h. Behavior test orders were randomized. A minimum of 18 h separated behavior testing in days one and two. Male animals were tested before females to avoid potential confounding effects of pheromones on behavior. Behavioral apparatuses were thoroughly cleaned with 70% ethanol between each animal. One hour following behavior testing on males, female behaviors were examined. All behavior studies were conducted under red lighting and recorded for later analysis.

#### Cage mate separation and ultrasonic vocalizations (USVs)

To conduct the cage mate separation test, cage mates were removed from their home cage and placed into a 50 × 25 × 30 cm aquarium and allowed to explore for 2 min [41, 64]. After 2 min, one cage mate was removed and placed into a separate aquarium. Each aquarium was enclosed within a sound dampening chamber equipped with an Avisoft UltraSoundGate 116Hb (CM16/CMPA) condenser microphone (Avisoft Bioacoustics, Nordbahn, Germany). USVs emitted for five minutes in response to the cage mate separation were recorded using Avisoft-Recorder USGH software (Avisoft Bioacoustics, Nordbahn, Germany). Separated cage mates will emit 50 kHz USVs, which are due to the separation and not the novelty of a new chamber [49]. This separation of cage mate protocol was used to avoid confounding effects of pheromones or hormones on USVs [44, 50–52, 65–67]. Recorded USVs were analyzed using Avisoft SASLab Pro (Version 5.2.12, Avisoft Bioacoustics, Nordbahn, Germany). USV spectrogram analyses were conducted using Avisoft recommended settings of fast Fourier transform (FFT)-length of 256 points and an overlap of 50%. Spectrograms had a frequency resolution of 0.977 kHz and a time resolution of 0.5 milliseconds (ms). A threshold between − 40 and − 50 decibels (dB) was chosen to remove background noise. If background noise interfered with USV classification, those USVs were not analyzed. Calls were manually selected within the spectrogram and categorized by call type [41, 45, 53, 64]. Experimenters analyzing USVs were blinded to the treatments.

**Fig. 2:**
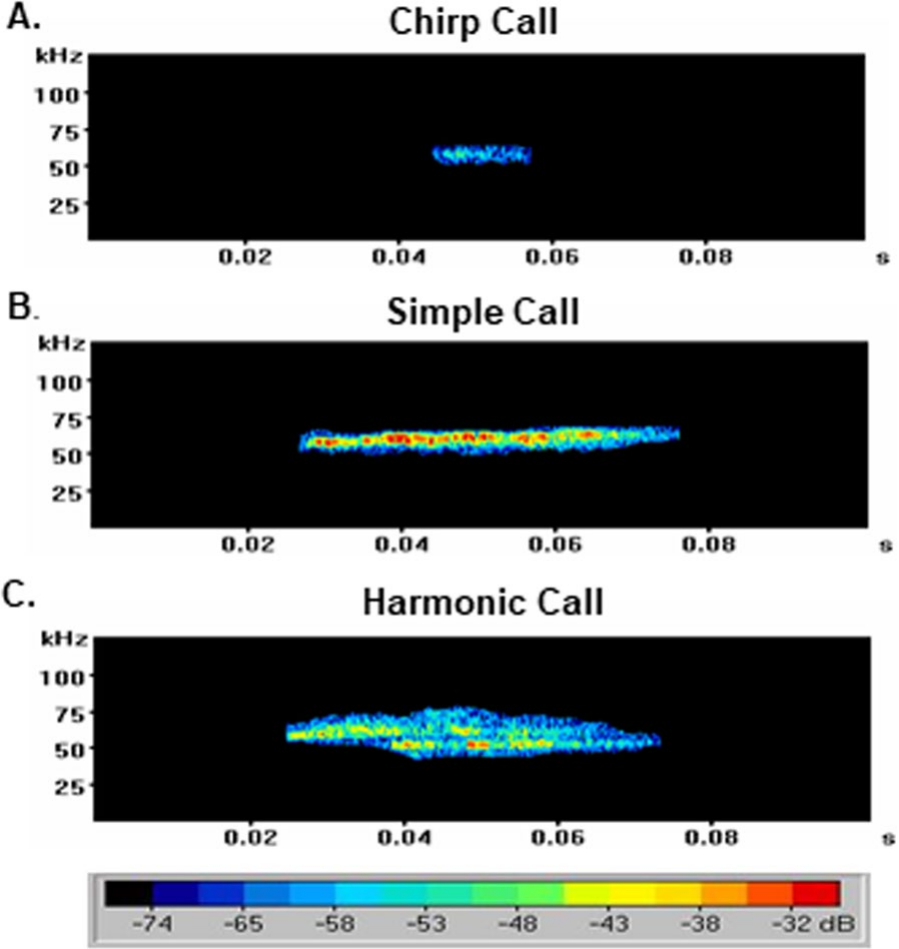
Representative spectrograms of ultrasonic vocalizations (USVs). USVs collected during a 5-min cage mate separation were analyzed. USVs were manually selected within spectrograms and categorized by call type. **A** Chirp calls were defined as low intensity USVs with a duration less than 0.02 s (20 ms). **B** Simple calls were defined as USVs longer than 0.02 s (20 ms) with “straight-line” intensity. **C** Harmonic calls were defined as a single USV with distinct tones of varying duration and intensity that create a “harmony”. Color-coded decibel level shown in key. kHz = kilohertz; s = seconds; dB = decibel

USVs were characterized by total call frequency (number of calls over the 5-min interval), call type (chirp, simple, harmonic), latency to first call, duration, intensity (dB), and bandwidth (kHz). USV intensity and bandwidth are included in analysis as impairments of these properties are linked with impaired nigrostriatal pathway function [36]. Stress calls (22 kHz) were not observed in this study. Therefore, all USVs in this study were emitted at the 50 kHz frequency, which are USVs produced during conditions of reward, positive affect, or normal behaviors. USVs were separated into chirp, simple, and harmonic call types based on their duration and intensity (Fig. 2). Chirp calls were defined as low intensity flat USVs with a duration less than 0.02 s (20 ms), whereas simple calls were defined as flat USVs lasting longer than 0.02 s. Harmonic calls were defined as a single call with two distinct tones, creating a ‘harmony’ with varying duration and intensity. USVs calls that could not be classified as chirp, simple, or harmonic calls, were classified as other calls and included in the total USV call analysis (Additional file 1).

### Open field test

Gross and fine motor activities were quantified using the San Diego Instruments Photobeam Activity System-Open field arena (40.64 × 40.64 × 38.1 cm) with a unidirectional “rearing” bar located above a bi-directional main field bar, as previously described [62]. Open field behaviors of distance traveled in meters (m), number of unassisted rears, and number of total rears were tracked and analyzed for a period of 10 min. An unassisted rear was classified when the animal stood on its hindlimbs without touching a wall or requiring aid to stand fully erect.

### Tissue sample collection and preparation

During the first 2 h of the animals’ wake phase, each animal was anesthetized with isoflurane (2–3%) and decapitated [60–63]. Trunk blood was collected in EDTA-coated tubes and centrifuged at 2000×*g* for 10 min at 4 °C to collect plasma, which was stored at − 80 °C.

Each brain was quickly removed, flash frozen in phosphate-buffer saline (PBS), sliced into 1-mm coronal sections using a brain matrix, and brain nuclei containing the substantia nigra (− 5.30 mm from Bregma) were isolated according to Paxinos and Watson’s brain atlas [68] using blunt 20-gauge needles attached to 1 mL syringes [60–62]. Micro-dissected brain samples were placed into microcentrifuge tubes to be stored at − 80 °C until protein analysis. Whole placental tissues were collected, weighed, and flash-frozen in liquid nitrogen before storing at − 80 °C on GD20. Prior to homogenization, the maternal decidua was removed from each placenta and the remaining tissue was used for immunoblotting.

### Circulating oxidative stress

Circulating oxidative stress in plasma proteins was assayed using OxiSelect Advanced Oxidative Protein Products (AOPP) kit (Cell Biolabs, Inc., San Diego, CA) according to our previously published protocol [60]. This assay measures the micromolar (µM) concentration of all oxidized proteins in the plasma relative to a known standard. Chloramine in the kit reacts with oxidized proteins to produce a color change, which can be read at 340 nm (nm).

### Western blot

Frozen tissue samples were homogenized in RIPA lysis buffer (VWR, cat # N653) containing (per 0.5 ml): 2.5 µl Halt™ protease and phosphatase inhibitor (Thermo Scientific, cat # 78442), 1 µl 0.5 M ethylenediaminetetraacetic acid (EDTA, Sigma-Aldrich), and 1 µl 0.5 mM dithiothreitol (DTT, Sigma-Aldrich), as previously described [60]. For total protein concentration determination, protein levels in the homogenate were determined using Pierce BCA Protein Assay (Thermo Fisher, cat # 23225). Samples were denatured with β-mercaptoethanol and boiled at 100 °C for 5 min. Equal volumes containing 15–30 µg protein were loaded into a Bio-Rad 4–15% polyacrylamide gel. Electrophoresis was conducted at 25 milliamps (mA) in a Tris–glycine running buffer followed by overnight transfer at 4 °C onto a PVDF membrane at 50 mA. Following 30 min washing, membranes were blocked for 30 min with 5% nonfat milk in Tris-based saline (TBS)–Tween (TBS-T) at room temperature. Membranes were then transferred to 1% nonfat milk TBS-T solutions containing specific primary antibodies (anti-tyrosine hydroxylase, Millipore AB318 1:1000; Anti-Spectrin, EMD Millipore MAB1622 1:1000) and incubated overnight at 4 °C. Primary antibody against beta actin (GeneTex GTX 629630, 1:5000) was incubated for 1 h at room temperature. Afterwards, membranes were washed in 10 min increments for 30 min, and then incubated in 1% milk TBS-T with secondary antibody solutions (HRP-conjugated goat anti-mouse, Invitrogen 31430, 1:5000) at room temperature for 1 h. Protein bands were visualized using West Pico enhanced chemiluminescence detection assay (Thermo Scientific, cat # 34580) on a Syngene G:Box system using FlourChem HD2 AIC software as previously described [69]. NIH Image J software (version 1.48v) was used to quantify band densitometry, and values were normalized to beta actin.

### Statistical analyses

Significance was defined as *p* < 0.05. Statistical analyses were conducted in Prism Version 8.4 (GraphPad, San Diego, CA). Outliers were identified and removed using the ROUT method (coefficient *Q* = 1%). Data distribution was tested using the Shapiro–Wilk test. For analysis of pubertal (PND 48) and young adult (PND 66) offspring outcomes, data with non-Gaussian distributions were normalized using square root transformation. Litter size, fetal and placental weights, and placental protein content were compared using an unpaired *t* test, while gestational length was compared using a Mann–Whitney *U* test. A two-way ANOVA (condition, sex) followed by Sidak's or Tukey’s posthoc test was used to analyze neonatal biometrics and pubertal and young adult behaviors and protein expression. Data are presented as mean with S.E.M, unless otherwise indicated.

## Results

### Gestational hypoxia during late gestation did not affect maternal, fetal, or offspring body weights

All timed pregnant rats were weighed (203–276 g) upon arrival at the University of North Texas Health Science Center. No differences were observed in dam weights prior to initiation of CIH (normoxia: 250.8 ± 5.7 g, CIH: 255.9 ± 6.47 g, *p* > 0.05). In study one, dams were sacrificed during the postpartum period, 28 days following CIH. In study two, dams were sacrificed on GD20, approximately 16 h following the last bout of CIH. Maternal postpartum body weights at euthanasia did not differ between groups (normoxia: 284.14 ± 16.46 g, CIH: 282.45 ± 19.04 g; *p* > 0.05). Similarly, CIH exposure did not impact maternal body weights on GD20 (normoxia: 344.67 ± 5.30 g, CIH: 328.81 ± 9.87 g; *p* > 0.05).

Gestational CIH did not affect litter size (normoxia: 11.5 ± 1.8, CIH: 9.7 ± 1.6; *p* > 0.05), number of resorptions (normoxia: 0.0 ± 0.0 vs. CIH: 0.5 ± 1.2; *p* > 0.05), fetal weights (normoxia: 2.48 ± 0.07 g, CIH: 2.37 ± 0.05 g; *p* > 0.05), placental weights (normoxia: 0.49 ± 0.02 g, CIH: 0.53 ± 0.02 g; *p* > 0.05), or placental efficiency as measured by fetoplacental weight ratios (normoxia: 5.07 ± 0.47 vs. CIH: 4.57 ± 0.54; *p* > 0.05) at GD20. Similarly, there were no differences in body weights between neonates from CIH-exposed pregnancies compared to normoxic pregnancies (Table 1). Gestational CIH did not affect pubertal (PND48) offspring body weights (Males, normoxia: 295.9 ± 6.72 g, CIH: 285.6 ± 7.70 g; *p* > 0.05*,* Females, normoxia: 220.4 ± 3.46 g, CIH: 222.5 ± 5.1 g; *p* > 0.05) or young adult (PND66) offspring body weights (Males, normoxia: 438.3 ± 5.98 g, CIH: 460.6 ± 8.35 g; *p* = 0.098; Females, normoxia: 287.5 ± 6.24 g, CIH: 276.8 ± 7.00 g; *p* > 0.05).

**Table 1:** Neonatal biometrics after birth

		Treatment	
		Normoxia	CIH
Neonatal weight (g)	Male	6.55 ± 0.23	6.63 ± 0.21
	Female	6.43 ± 0.20	6.42 ± 0.23
Crown-rump length (cm)	Male	4.08 ± 0.08	4.05 ± 0.05
	Female	4.06 ± 0.08	4.06 ± 0.08
Abdominal girth (cm)	Male	4.82 ± 0.12	4.75 ± 0.12
	Female	4.70 ± 0.10	4.74 ± 0.06

Means ± SEM. Two-way ANOVA with Sidak's multiple comparison test (*n* = 8/group). All comparisons had *p*-values > 0.05. *CIH*  chronic intermittent hypoxia

Gestational length was greater in the CIH group compared to control, but this difference was not statistically significant (median (IQR), normoxia: 22 (22–23) days; CIH: 23 days (23–23); *p* = 0.06). There were no differences in number of live births between groups (normoxia: 12.38 ± 1.27, CIH: 10.63 ± 1.05; *p* > 0.05).

### Gestational hypoxia reduced total calls emitted by pubertal offspring and increased latency to first call in young adult male offspring

**Fig. 3:**
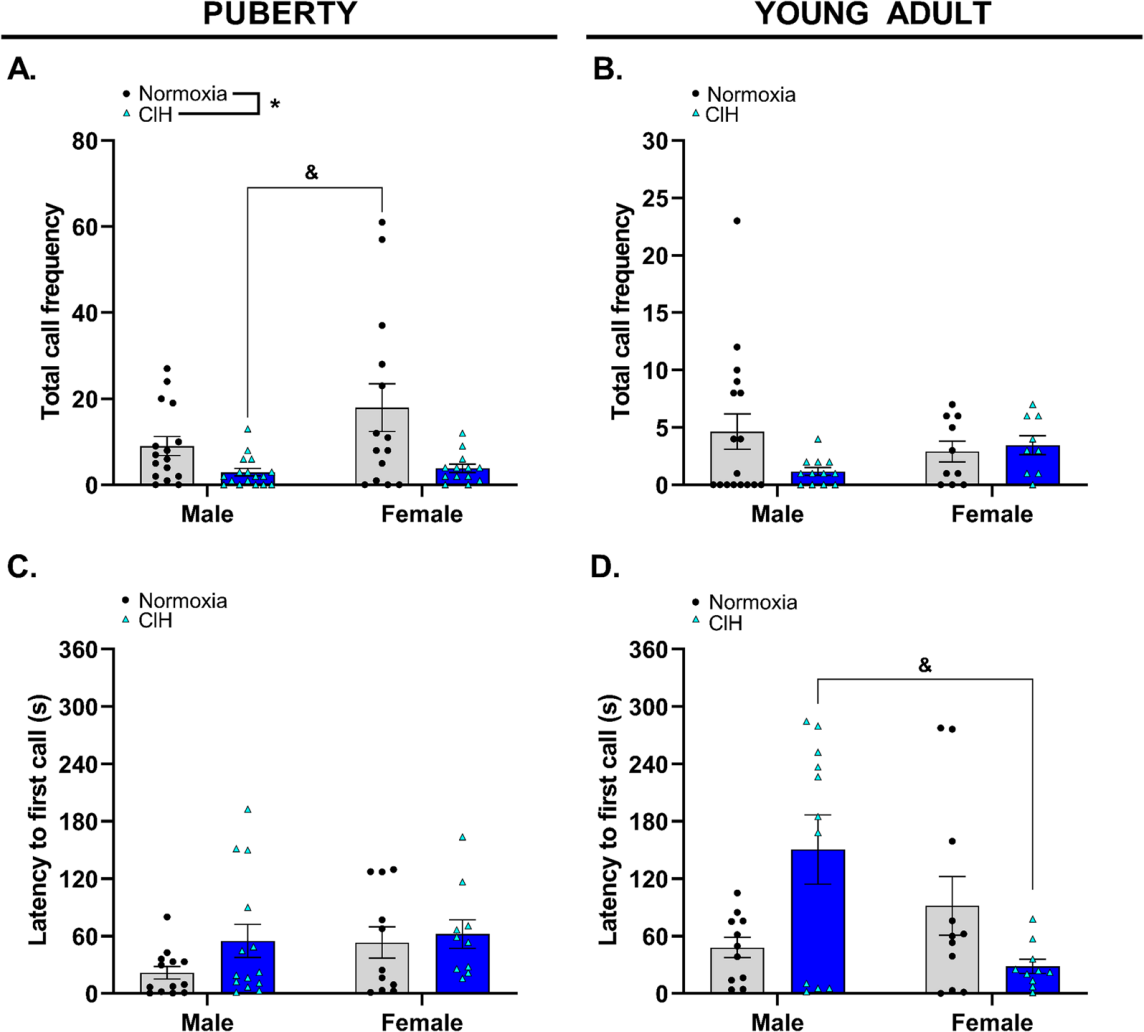
Total call frequency and latency to first call. Total call frequency (**A**, **B**) and latency to first call (**C**, **D**) produced by pubertal (**A**, **C**) and young adult (**B**, **D**) male and female offspring exposed to normoxia or chronic intermittent hypoxia (CIH) in utero. Analyzed by Two-way ANOVA with Tukey’s multiple comparisons test, *n* = 9–17/group. Frequency = number of calls per 5-min interval; s = seconds. * = main effect of CIH; & = Tukey’s comparison. *, & = *p* < 0.05

Gestational hypoxia reduced the frequency of total USVs (all call types) emitted by pubertal offspring (*p* = 0.002, Fig. 3A, Additional file 1). Female pubertal offspring from normoxic pregnancies emitted significantly more calls compared to male pubertal offspring exposed to gestational CIH. There was no effect of gestational CIH on total number of calls emitted by male or female young adult offspring (*p* > 0.05, Fig. 3B). However, young adult offspring from normoxic pregnancies emitted fewer calls compared to pubertal offspring from normoxic pregnancies (*p* = 0.004), with female young adult offspring emitting significantly fewer calls compared to female pubertal offspring (*p* = 0.018). Although gestational CIH reduced total call frequency in pubertal offspring, there was no effect of gestational CIH on latency to first call in male or female pubertal offspring (*p* = 0.089, Fig. 3C). In young adults, the effect of gestational CIH on latency to first call was dependent on sex (*p* = 0.018), with an increase in latency to first call in CIH-exposed young adult male offspring compared to CIH-exposed young adult females (Fig. 3D).

### Gestational hypoxia impacted chirp calls in pubertal and young adult offspring

**Fig. 4:**
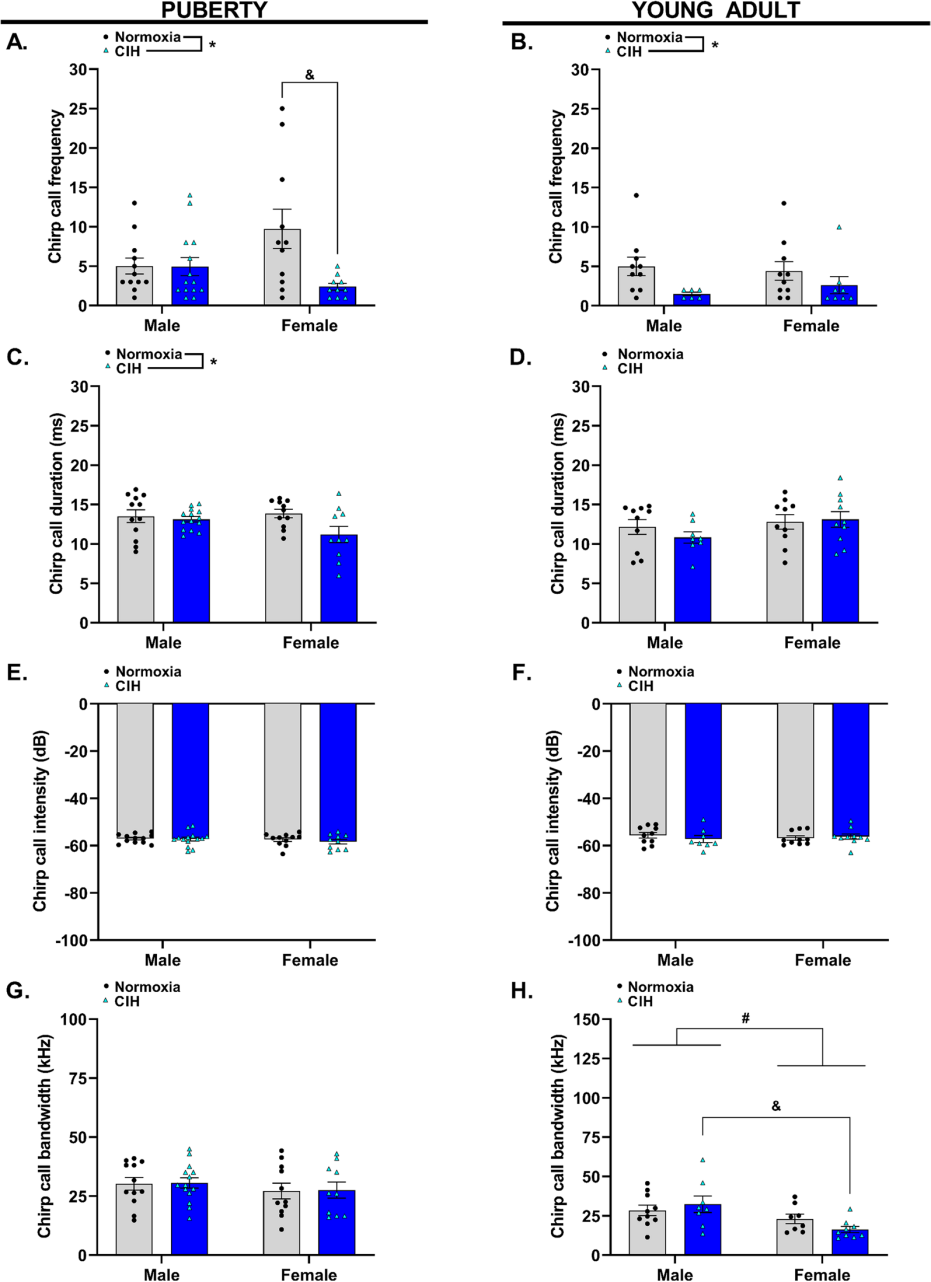
Characterization of pubertal and young adult offspring chirp calls. Frequency (**A**, **B**), duration (**C**, **D**), intensity (**E**, **F**), and bandwidth (**G**, **H**) of chirp calls emitted by pubertal (**A**, **C**, **E**, **G**) and young adult (**B**, **D**, **F**, **H**) male and female offspring exposed to normoxia or chronic intermittent hypoxia (CIH) in utero. Analyzed by Two-way ANOVA with Tukey’s multiple comparisons test, *n* = 8–14/group. Frequency = number of calls per 5-min interval, ms = milliseconds, dB = decibels, kHz = kilohertz. * = main effect of CIH; # = main effect of sex; & = Tukey’s comparison; *, #, & = *p* < 0.05

Since gestational CIH decreased the total number of offspring vocalizations, we categorized the calls to determine if these differences were attributed to a specific call type. Chirp calls were defined as low-intensity USVs with a duration less than 20 ms (Fig. 2). Gestational hypoxia reduced frequency of chirp calls emitted by pubertal offspring (*p* = 0.010, Fig. 4A, Additional file 1), and this effect was also present in young adult offspring (*p* = 0.010, Fig. 4B). The effects of gestational CIH on chirp call frequency in pubertal offspring were dependent on sex (*p* = 0.021, Fig. 4A). Specifically, female pubertal offspring exposed to gestational CIH emitted fewer chirp calls compared to pubertal females from normoxic pregnancies. There was no effect of gestational CIH on frequency of chirp calls emitted by pubertal males compared to age-matched males from normoxic pregnancies. Moreover, reductions in chirp calls were not observed in young adult females exposed to gestational CIH compared to young adult females from normoxic pregnancies (Fig. 4B). Gestational CIH did not affect chirp call duration in young adult offspring (*p* > 0.05, Fig. 4D) but reduced the duration of chirp calls emitted by pubertal offspring (*p* = 0.032, Fig. 4C). Although gestational CIH affected chirp call frequency and duration, CIH exposure did not impact the intensity of chirp calls emitted by pubertal offspring (*p* > 0.05, Fig. 4E). Moreover, gestational CIH did not affect chirp call intensity in young adult offspring (*p* > 0.05, Fig. 4F). In addition, gestational CIH did not affect chirp call bandwidth in pubertal offspring (*p* > 0.05, Fig. 4G). Nonetheless, there was a sex effect on chirp call bandwidth in young adult offspring, with males emitting chirp calls of significantly increased bandwidth compared to females (*p* = 0.005, Fig. 4H). Specifically, young adult males exposed to gestational CIH emitted chirp calls of greater bandwidth than CIH-exposed young adult females.

### Pubertal female offspring exposed to gestational hypoxia emit fewer simple calls

**Fig. 5:**
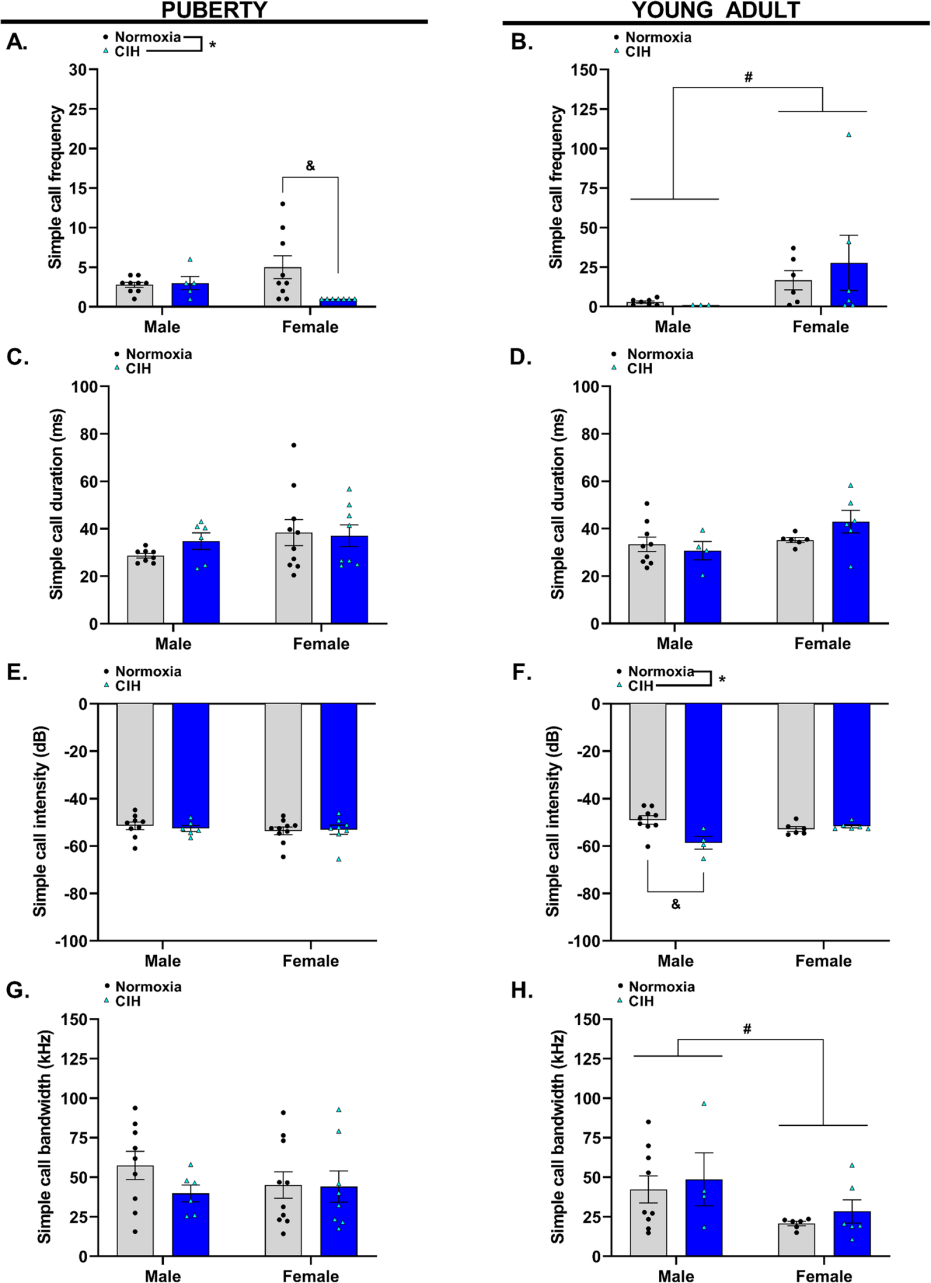
Characterization of pubertal and young adult offspring simple calls. Frequency (**A**, **B**), duration (**C**, **D**), intensity (**E**, **F**), and bandwidth (**G**, **H**) of simple calls emitted by pubertal (**A**, **C**, **E**, **G**) and young adult (**B**, **D**, **F**, **H**) male and female offspring exposed to normoxia or chronic intermittent hypoxia (CIH) in utero. Analyzed by Two-way ANOVA with Tukey’s multiple comparisons test, *n* = 4–10/group. Frequency = number of calls per 5-min interval, ms = milliseconds, dB = decibels, kHz = kilohertz. * = main effect of CIH; # = main effect of sex; & = Tukey’s comparison; *, #, & = *p* < 0.05

Simple calls were defined as USVs of greater than 20 ms duration that can be visibly seen as a straight line (Fig. 2). Gestational CIH reduced simple calls emitted by pubertal offspring (*p* = 0.028), and this effect was dependent on sex (*p* = 0.022, Fig. 5A, Additional file 1). Precisely, CIH-exposed pubertal females emitted fewer simple calls than female pubertal offspring from normoxic pregnancies. Young adult females emitted more simple calls than young adult males (*p* = 0.024); however, there was no effect of gestational CIH on the production of simple calls by young adult offspring (*p* = 0.914 Fig. 5B). There were no effects of gestational CIH on simple call duration in pubertal (*p* > 0.05, Fig. 5C) or young adult offspring (*p* > 0.05, Fig. 5D). Although not statistically significant, female young adult offspring emitted simple calls with greater duration than male young adult offspring (*p* = 0.063, Fig. 5D).

There was no effect of gestational CIH on intensity of simple calls emitted by pubertal offspring (*p* > 0.05, Fig. 5E). However, CIH exposure reduced the intensity of simple calls emitted by young adult offspring (*p* = 0.022), and this effect was dependent on sex (*p* = 0.005, Fig. 5F). Specifically, CIH-exposed young adult males emitted simple calls with lesser intensity (decreased decibels) compared to young adult offspring from normoxic pregnancies. Similar to pubertal simple call duration and intensity, gestational CIH had no effect on the bandwidth of simple calls emitted by pubertal offspring (*p* > 0.05, Fig. 5G). Moreover, there was no effect of gestational CIH on bandwidth of simple calls emitted by young adult offspring (*p* > 0.05, Fig. 5H). Yet, young adult males emitted simple calls with greater bandwidths compared to young adult female offspring (*p* = 0.034).

### Gestational hypoxia increased complexity of harmonic calls in young adult males

**Fig. 6:**
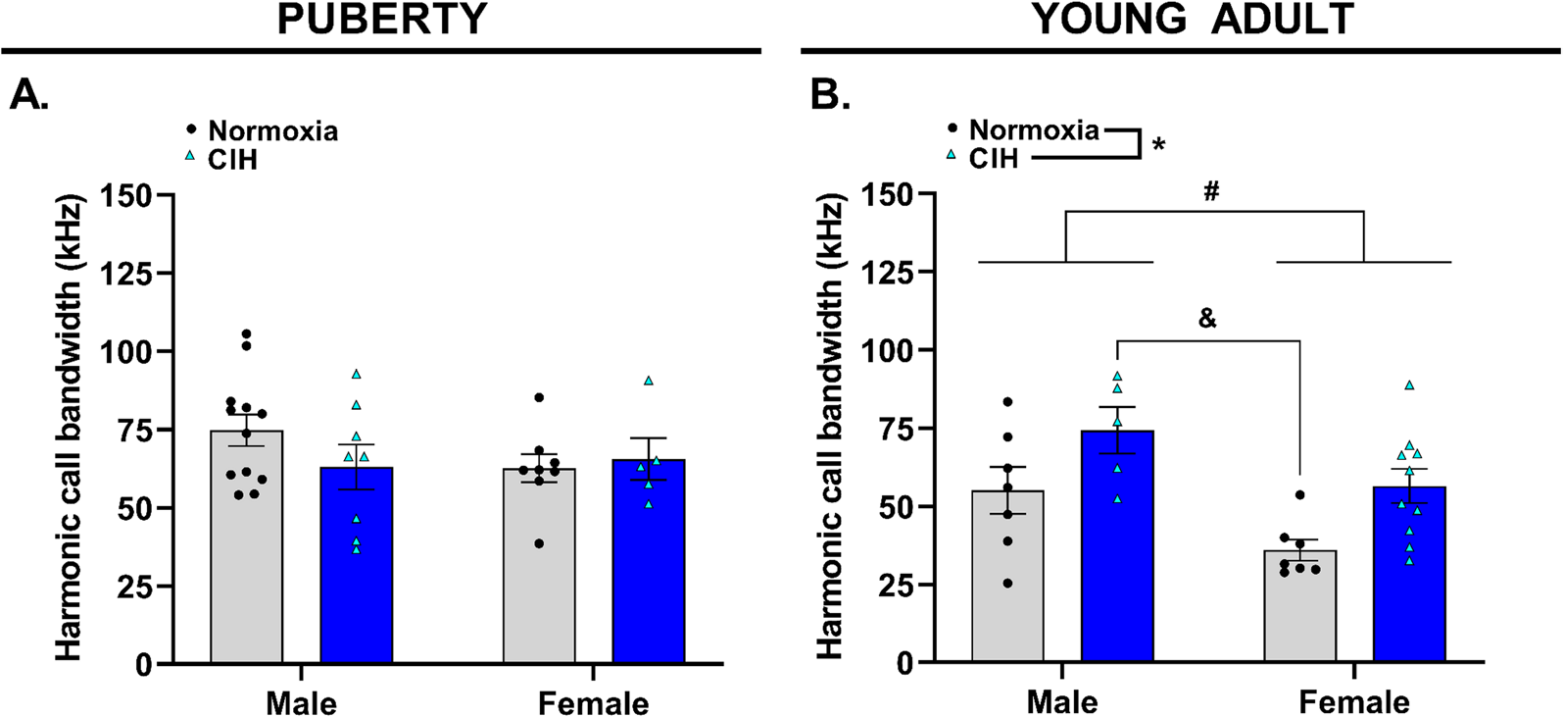
Bandwidth of harmonic calls emitted by pubertal and young adult offspring. Bandwidth of harmonic calls produced by (**A**) pubertal and (**B**) young adult male and female offspring exposed to normoxia or chronic intermittent hypoxia (CIH) in utero. Analyzed by Two-way ANOVA with Tukey’s multiple comparisons test, *n* = 5–12/group. kHz = kilohertz. * = main effect of CIH; # = main effect of sex, & = Tukey’s comparison; *, #, & = *p* < 0.05

Harmonic calls are USVs with two separate, yet identifiable tones, creating a ‘harmony’ (Fig. 2). In pubertal offspring, there was no effect of gestational CIH on frequency of harmonic calls (*p* > 0.05), harmonic call duration (*p* > 0.05), harmonic call intensity (*p* > 0.05), or harmonic call bandwidth (*p* > 0.05, Fig. 6A, Additional file 1). Similarly, gestational CIH did not affect young adult offspring frequency of harmonic calls (*p* > 0.05), harmonic call duration (*p* > 0.05), or harmonic call intensity (*p* > 0.05). However, young adult males produced harmonic calls with greater bandwidths compared to young adult female offspring (*p* = 0.007, Fig. 6B). Additionally, harmonic call bandwidth was greater in CIH-exposed young adult offspring compared to young adult offspring from normoxic pregnancies (*p* = 0.004). Specifically, CIH-exposed male young adults emitted harmonic calls with greater bandwidths than young adult females from normoxic pregnancies.

### Gestational hypoxia did not impact offspring motor function

**Fig. 7:**
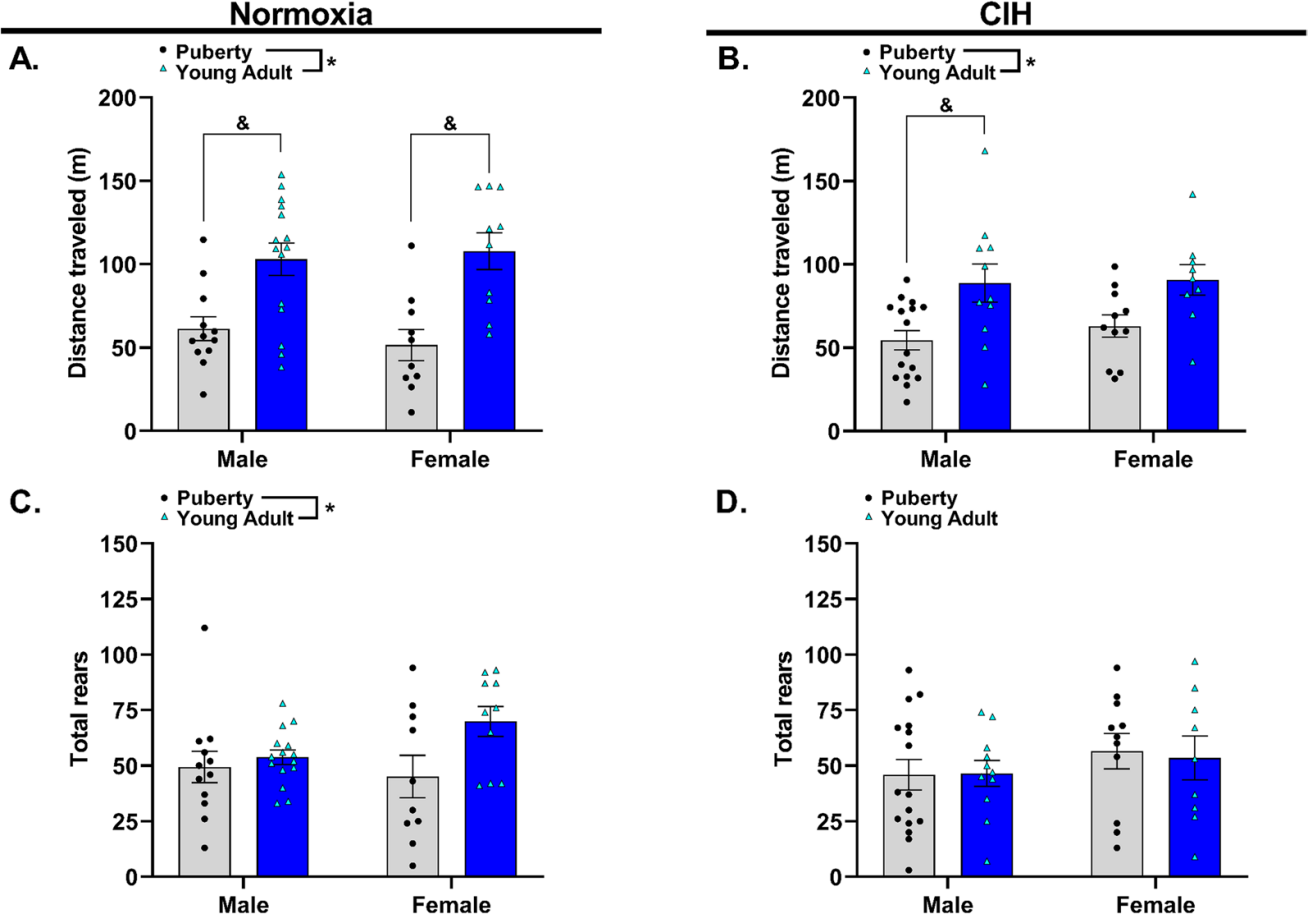
Distance traveled and total rearing behavior by pubertal and young adult offspring during modified open field behavior test. Total distance traveled (**A**, **B**) and total rears (**C**, **D**) by pubertal and young adult male and female offspring exposed to normoxia (**A**, **C**) or chronic intermittent hypoxia (CIH, **B**, **D**) in utero. Analyzed by Two-way ANOVA with Tukey’s multiple comparisons test, *n* = 9–16/group. kHz = kilohertz. * = main effect of CIH, & = Tukey’s comparison; *, & = *p* < 0.05

Gross and fine motor skills were assessed using an open field behavior test. Gross motor function was quantified by examining distanced traveled (meters) and total rearing behaviors, while fine motor function was assessed by observing unassisted rearing behaviors during a 10-min test. No significant differences were observed in pubertal animals in distance traveled regardless of sex or exposure to gestational CIH (*p* > 0.05). Similarly, no significant differences were observed in distance traveled by young adult males or females (*p* > 0.05). Distance traveled was significantly greater in young adult offspring from normoxic pregnancies compared to pubertal offspring from normoxic pregnancies (*p* < 0.0001), and this effect was significant for males and females (Fig. 7A). Distance traveled was significantly greater in young adult offspring from CIH exposed pregnancies compared to pubertal offspring from CIH exposed pregnancies (*p* = 0.0006). This effect was significant for males and not for females (Fig. 7B).

Total rearing behaviors were also assessed to examine gross motor behavior. Total rears were significantly greater in young adult offspring from normoxic pregnancies than pubertal offspring from normoxic pregnancies (*p* = 0.031) (Fig. 7C). Gestational CIH did not impact total rearing behavior, regardless of sex or age (*p* > 0.05) (Fig. 7D). When assessing fine motor function, no impact of age or sex was observed on unassisted rears in offspring from normoxic pregnancies (MN-puberty: 12.42 ± 3.32, MN-young adult: 14.13 ± 2.09, FN-puberty: 7.10 ± 2.12, FN-young adult: 13.30 ± 2.01; *p* > 0.05). Similarly, there was no effect of gestational CIH on unassisted rears by young adults or pubertal rats (MC-puberty: 9.13 ± 1.95, MC-young adult: 12.91 ± 1.91, FC-puberty: 9.00 ± 1.84, FC-young adult: 7.44 ± 2.44; *p* > 0.05).

### Gestational hypoxia increased oxidative stress in young adult offspring

**Fig. 8:**
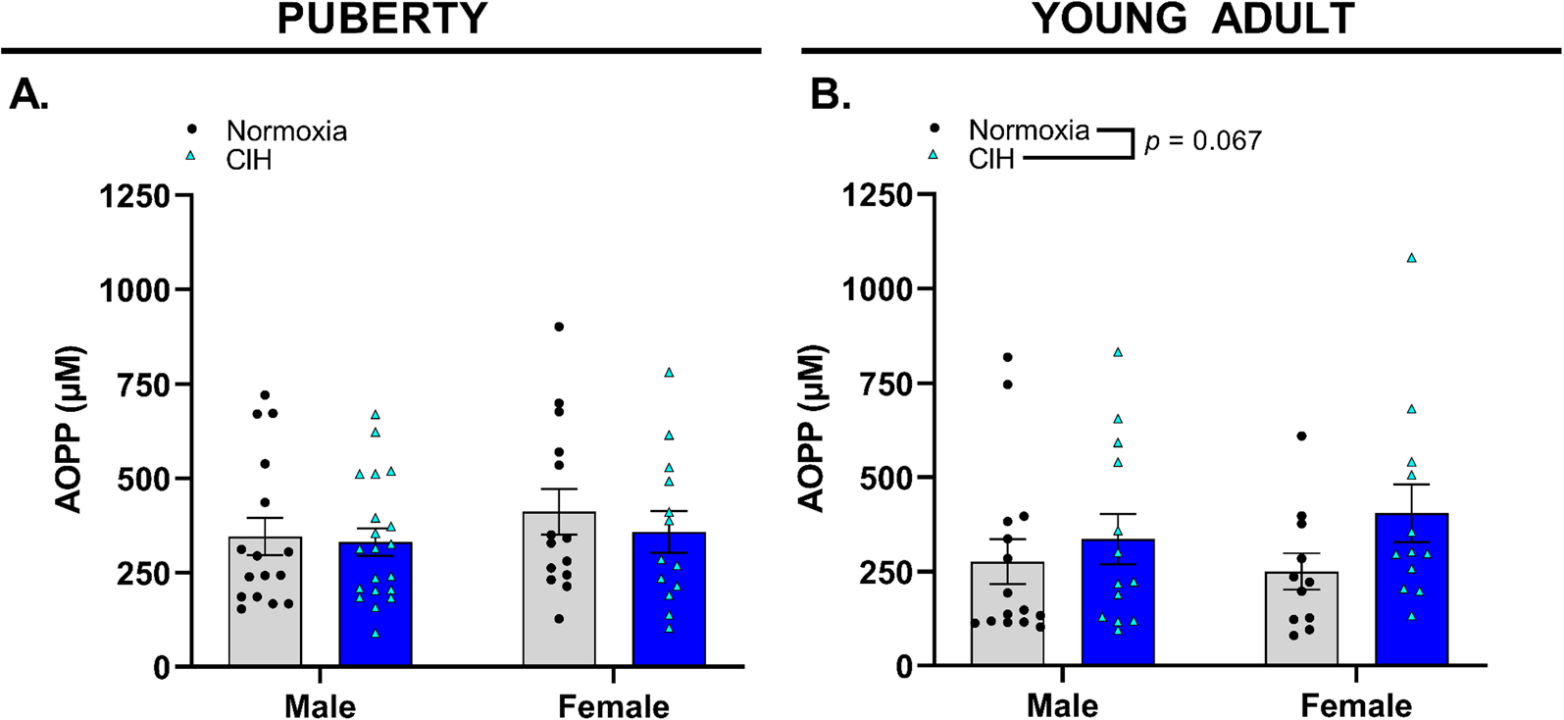
Circulating advanced oxidation protein products (AOPP) in rat offspring plasma. Concentration of plasma oxidative stress marker, AOPP, in (**A**) pubertal and (**B**) young adult male and female offspring exposed to normoxia or chronic intermittent hypoxia (CIH) in utero. Analyzed by two-way ANOVA with Tukey’s multiple comparisons test, *n* = 10–20/group

Since we observed CIH-induced deficits in pubertal and young adult offspring USVs, we examined markers of oxidative stress within the circulation and in specific brain regions associated with USVs and motor function. Circulating oxidative stress (AOPP) was not increased in pubertal offspring regardless of sex or exposure to gestational CIH (*p* > 0.05, Fig. 8A). Circulating oxidative stress was not increased in young adult offspring exposed to gestational CIH compared to age matched offspring from normoxic pregnancies, (*p* = 0.067, Fig. 8B).

In many neurological pathologies, damage to neurons precedes onset of symptoms. Abnormal proteolysis of cytoskeletal proteins, such as spectrin, by proteases including calpains and caspases contributes to neuronal cell damage or even death [70, 71]. Thus, we examined calpain cleavage (oxidative stress-associated mechanisms) and caspase-3 cleavage (apoptosis-associated mechanisms) of spectrin in the substantia nigra, a brain region associated with rodent USVs and motor function (Table 2) [34–36]. Within the substantia nigra of pubertal and young adult offspring, no significant differences were observed in calpain cleavage of spectrin regardless of sex or exposure to gestational CIH (*p* > 0.05). Likewise, there were no observed effects (*p* > 0.05) of sex or gestational CIH on caspase-3 cleavage of spectrin, an indicator of apoptosis [70, 71], in the substantia nigra of pubertal and young adult offspring. Since prior studies found that loss of dopaminergic neurons in the substantia nigra impaired USVs [36, 72, 73], we assessed if gestational CIH at late gestation impacted dopaminergic neuron expression in the substantia nigra. We used a marker of dopaminergic neurons, tyrosine hydroxylase, to ascertain whether dopaminergic neurons within the substantia nigra were damaged (Table 2). There were no significant differences in tyrosine hydroxylase expression within the substantia nigra of pubertal and young adult offspring, regardless of sex or gestational CIH (*p* > 0.05). To assess whether gestational CIH during late gestation induced damage to placental tissue, we also examined spectrin cleavage by calpain and caspase-3 within placentas isolated on GD20 (Table 2). There was no effect of CIH exposure on calpain or caspase-3 cleavage of placental spectrin (*p* > 0.05).

**Table 2:** Protein expression analysis of damage-associated markers in substantia nigra and placenta

		Substantia nigra				Placenta	
		Puberty		Young adult		GD20	
		Normoxia	CIH	Normoxia	CIH	Normoxia	CIH
Calpain	M	163.3 ± 28.44	180.6 ± 18.09	204.3 ± 48.88	148.4 ± 38.99	123.2 ± 21.43	115.9 ± 22.57
	F	137.4 ± 31.07	165.2 ± 18.14	168.6 ± 17.66	232.2 ± 45.08		
Caspase-3	M	51.68 ± 12.72	60.75 ± 13.19	75.23 ± 24.04	41.47 ± 11.15	46.68 ± 8.11	47.96 ± 13.00
	F	46.55 ± 18.38	40.16 ± 9.60	70.15 ± 12.70	66.31 ± 14.90		
TH	M	108.9 ± 25.36	143.0 ± 53.12	106.9 ± 40.22	154.4 ± 47.87	Not examined	
	F	155.4 ± 43.11	98.58 ± 32.69	69.44 ± 22.75	151.9 ± 39.41		

Values represent percentage of Beta Actin expression and are displayed as means ± SEM. Two-way ANOVA with Tukey’s multiple comparison test for substantia nigra comparisons (*n* = 5–6/group). Unpaired *t* test for placenta comparisons (*n* = 6/group). No significant differences in any comparisons (*p* > 0.05). *GD*  gestational day, *CIH*  chronic intermittent hypoxia, *M*  male, *F*  female, *TH*  tyrosine hydroxylase

## Discussion

The hypothesis for this study was that exposure to hypoxia during late gestation would impair fetal brain maturation in a sex-dependent manner. The major findings are (1) brain maturation of the nigrostriatal pathway is sex- and age-dependent in rats and (2) hypoxia during late gestation impacts rat brain maturation of the nigrostriatal pathway that can be observed during puberty and young adulthood in offspring.

We observed that hypoxic stress during late pregnancy impacted brain maturation of the nigrostriatal pathway that can be observed during puberty and young adulthood but did not impact the structural development of the nigrostriatal pathway. Specifically, gestational CIH impacted brain maturation in female offspring more than male offspring during puberty: (a) decreased chirp USV call frequency and (b) decreased simple USV call frequency. In contrast, brain maturation in male offspring during young adulthood was more impacted than female offspring: (a) increased latency (or time) to first USV and (b) decreased simple call intensity. Interestingly, late gestational CIH quantitatively impaired (ability to make and sustain a call) the emission of USVs by females only during puberty, whereas CIH qualitatively impaired (USV call characteristics) the emission of USVs by adult males. It should be noted that the meaning of the different USVs is still relatively unknown. In this study, we used the cage mate separation protocol to elicit USVs, which is associated with re-establishing social contact. It is possible that late gestational hypoxia could have different effects on USV call characteristics in different environments, such as during sex behavior interactions. Although this is the first study to examine the impact of gestational CIH (GD 15–19) on USVs emitted by offspring during puberty and young adulthood, our findings are consistent with studies in humans that found prenatal hypoxic stress has more long-term effects in male offspring than female offspring [27, 28]. However, the literature on sex differences in response to gestational hypoxia in rodents is unclear, wherein there is a range of responses from differences in pregnancy outcomes, neonatal biometrics, and offspring physiology (e.g., oxidative stress, motor function).

Most gestational hypoxia studies use a long-term hypoxia protocol (> 12 days exposure) that encompasses all three gestational stages of pregnancy (Table 3), which is consistent with brain development in humans [74–76]. Brain developmental stages include (1) stage one wherein the neural progenitor cells become differentiated and the neural tube is formed [76, 77], (2) stage two when the cortical and subcortical regions of the brain are established with neural production [11–16, 77], and (3) stage 3 when maturation of brain cortical and subcortical pathways occur [16, 18, 77]. The effects of long-term gestational hypoxia on pregnancy outcomes or neonatal biometrics are generally dependent on the hypoxia protocol used (sustained hypoxia versus intermittent hypoxia cycles), in which sustained hypoxia is more likely to impact pregnancy outcomes and neonatal biometrics (Table 3). Similarly, the sustained hypoxia protocol negatively affects pregnancy outcomes and neonatal biometrics in shorter hypoxia protocols (6–9 days) that are restricted to brain development stages of 2–3. Based on findings that show no effects of acute sustained hypoxia protocols (< 1 day) during the final day of gestation on pregnancy outcomes and neonatal biometrics, development stages 2–3 are hypothesized to be the most important developmental stages for pregnancy outcomes and neonatal biometrics. Using an intermittent hypoxia cycling protocol that was restricted to GD 15–19 during brain developmental stage 3, our study focused on brain pathway maturation of the nigrostriatal subcortical pathway in offspring in the absence of negative effects on the process of placentation and having minimal impact on pregnancy outcomes and neonatal biometrics (Table 3).

**Table 3:** Comparison of gestational hypoxia parameters on offspring outcomes

Long-term gestational hypoxia (≥ 12 days): brain developmental stages 1–3						
Species	Gestational exposure	O_2_ (%)	Hypoxia (hours/day)	Hypoxic cycles from 21% O_2_	Impact on offspring	Citations
Rat (SD)	GD 5–21; 17 days	10%	8 h sleep phase	20 cycles/hour	*Age effects:* At birth: Decreased birth weight Puberty: No effect on body weight Adult: No effect on body weight	[115]
Rat (SD)	GD 3–19; 17 days	4%	8 h sleep phase	60 cycles/hour	*Age effects:* At birth: No effect on litter size or sex distribution Puberty: Decreased body weight Adult: Decreased body weight, increased myocardial SOD1 *Sex differences:* Adult: Increased myocardial lipid peroxidation in hypoxic males	[116]
Rat (SD)	GD 10–21; 12 days	10.5%	8 h sleep phase	15 cycles/hour	*Age effects:* At birth: No effect on litter size or body weight. No oxidative stress in placenta or fetal brains. Increased maternal separation-induced USVs at PND 4 Adult: No effects on open field locomotor behavior *Sex differences:* At birth: Increased maternal separation-induced harmonic calls in hypoxic females	[117]
Rat (SD)	GD 7–21; 15 days	10%	3 h	Sustained	*Age effects:* At birth: Decreased birth weight Puberty: No effect on body weight Adult: No effect on body weight	[118]
Rat (SD)	GD 7–21; 15 days	10%	3 h	Sustained	*Age effects:* At birth: No effect on litter size, decreased birth weight	[119]
Rat (Wistar)	GD 6–20; 15 days	13%	24 h	Sustained	*Age effects:* At birth: Increased placental weight, no effects on birth weight, fetal biometrics, litter size, or sex ratio. No oxidative stress or lipid peroxidation in hypoxic placentas	[120]
Mouse (C57BL/6 J)	GD 6–18; 13 days	14%	24 h	Sustained	*Sex differences:* Adult: Increased oxidative stress in hypoxic males but decreased oxidative stress in hypoxic females	[121]

Mid-term gestational hypoxia (6–9 days): brain developmental stages 2–3						
Species	Gestational exposure	O_2_ (%)	Hypoxia (hours/day)	Hypoxic cycles from 21% O_2_	Impact on offspring	Citations
Rat (SD)	GD 15–20; 6 days	11%	24 h	Sustained	*Sex differences:* Adult: Decreased body weight in hypoxic males	[122]
Mouse (C57BL/6 J)	GD 10–18; 9 days	10.5%	24 h	Sustained	*Age effects:* At birth: Decreased litter size, crown to rump length, and abdominal circumference. No effects on placental weight or placental oxidative stress	[123]

Short-term gestational hypoxia (2–5 days): brain developmental stage 3						
Species	Gestational exposure	O_2_ (%)	Hypoxia (hours/day)	Hypoxic cycles from 21% O_2_	Impact on offspring	Citations
Rat (Long-Evans)	GD 15–19; 5 days	10%	8 h—sleep phase	10 cycles/hour	*Age effects:* At birth: No effect on litter size, body weights, placental weights, crown–rump length, or abdominal girth. Increased gestational length Puberty: Decreased USV calls. No effect on locomotor behavior (distance traveled or rearing) Adult: Increased bandwidth of harmonic USVs and increased oxidative stress. No effect on locomotor behaviors (distance traveled or rearing) *Sex differences:* Puberty: Decreased chirp USVs, chirp duration, decreased simple USVs in hypoxic females Adult: In males—increased latency to first USV and harmonic USV bandwidth with decreased chirp USVs and simple call USV intensity. In females—increased simple USV duration	Current Study
Mouse (CD-1)	GD 14–17; 4 days	11%	24 h	Sustained	*Age effects:* At birth: Decreased birth weight. No change in placental weights or litter size	[124]

Acute gestational hypoxia (< 1 day): brain developmental stage 3						
Species	Gestational exposure	O_2_ (%)	Hypoxia (hours/day)	Hypoxic cycles from 21% O_2_	Impact on offspring	Citations
Mouse (C57BL/6 J)	GD 17	9%	2 h	Sustained	*Age effects:* At birth: Increased brain weight Puberty: Increased volume of ventral tegmental area, pontine central grey matter, and cerebellar white matter (PND 23) Adult: No differences in brain volume	[125]
Mouse (C57BL/6 J)	GD 17.5	5%	2, 4, 6, or 8 h	Sustained	*Age effects:* At birth: No difference in litter size or gestational length. Decreased body weight that normalized at PND 8. No differences in brain apoptosis (cortex, basal ganglia, white matter) Adult: No differences in body weight, lateral ventricle size, anterior cingulate cortical thickness, or corpus callosum white matter. Decreased hindlimb strength	[126]

*SD*  Sprague–Dawley, *GD*  gestational day, *PND*  postnatal day, *USV*  ultrasonic vocalizations, *SOD1*  superoxide dismutase-1

Brain regions that are sensitive to prenatal hypoxia include the cortex, basal ganglia (includes the nigrostriatal pathway), and white matter in children [78]. The nigrostriatal pathway is integral for many behaviors, such as motor function, cognition, social interactions, mood (anxiety/depression), reward, and attention [79–81]. Therefore, impairments of the nigrostriatal pathway can have long-term consequences, such as many neuropsychiatric disorders (e.g., Parkinson’s disease [78, 82], addiction [78], cognitive dysfunction [78], autism spectrum disorders [78, 79], mood disorders [78, 79, 81], and attention deficit hyperactivity disorders [80]). These relationships have also been observed in clinical studies examining children exposed to gestational hypoxia (pre-eclampsia, acute perinatal hypoxic injury, and gestational sleep apnea [1–5]). Preeclampsia is associated with autism spectrum disorders, attention deficit disorders, schizophrenia, mood disorders, developmental delay, cognitive dysfunction, stroke, cerebral palsy, and brain connectivity [83–94]. Similarly, gestational sleep apnea is associated with neuropsychiatric outcomes in children, such as social interactions [95]. Interestingly, gestational sleep apnea impacts male children more than female children with respect to social interactions, cognitive function, and communication [96], which is consistent with the findings from our study. Moreover, the hypoxic protocol used in the current study is consistent with gestational sleep apnea, in which the hypoxic stress is intermittent and only present during the sleep period.

In addition to gestational CIH effects, we observed age- and sex-related developmental effects on brain maturation. Young adult males from normoxic pregnancies emitted USV calls (all call types) at greater bandwidths than young adult females, which is in agreement and extends prior findings showing male mice emitted larger bandwidths in complex calls than female mice [67]. Further, we showed that pubertal rats emitted more USVs than young adult rats. Prior studies have not compared pubertal USV call frequencies to young adult USV call frequencies, much less USV call frequencies in females. However, a recent study comparing USV call frequency between adult male C57BL/6 J mice at various ages (PND 56, PND 140, and PND 210) found that USV call frequencies declined with age in sexually naïve male mice, whereas this relationship with age was not observed in sexually experienced male mice [97]. Although pubertal C57BL/6 J mice (~ PND 30) were not included in this study [97], it is probable that age is the main factor for the observed decline in USV call frequency observed in our findings between sexually naïve young adult rats and pubertal rats.

Interestingly, our study demonstrates young adult females vocalize more simple USV calls compared to males, with no sex differences observed in other USV call frequencies (total USVs, chirp USVs, harmonic USVs). This is in contrast to findings from several other studies that found males emit more USVs than females [45, 65, 67, 98, 99]. It should be noted that these other studies were conducted under different parameters than the current study, in which USVs were quantified during male–female interactions. Changing the parameters or the environment that USVs are collected can dramatically impact USVs. Indeed, no sex differences in USVs are observed under conditions of same-sex interactions in mice [100]. Female mice emit more USVs in the presence of novel female conspecifics than familiar female conspecifics [101]. To remove the potential confounds of sexual experience, hormonal conditions, or novelty from conspecific animals that can independently impact USVs [44, 50–52, 65–67], we used the cage mate separation protocol to elicit USVs [49]. The cage mate separation protocol to elicit USVs has only been used in male rats [49, 54], but our results show that this protocol is sufficient to also elicit USVs in female rats. This cage mate separation protocol resulted in unmasking a previously unknown sex difference in which young adult female rats vocalize more simple USVs than young adult male rats.

Additionally, we demonstrated sex- and age-dependent differences in motor activity. Specifically, young adult male and female rats exhibited greater locomotor activity (distance traveled) than pubertal male and female rats. Young adult females displayed increased total rearing behaviors compared to pubertal females. Our results on motor function are consistent with prior reports in the literature. Several studies have reported increased total rearing behaviors in young adult female rats compared to pubertal female rats, young adult male rats, and pubertal male rats [102–105].

In contrast to the literature on total rearing behaviors, previous studies on sex differences in locomotion are equivocal. Several studies show increased locomotor activity in young adult male and female rats compared to pubertal male and female rats [102, 106–109], which is consistent with our results. Other studies have shown increased locomotor activity in female rats compared to male rats, whereas others reported no sex differences in locomotor activity in rats that range in age from PND 30–PND 80 [110, 111]. These differences in locomotor activity could be due to environmental factors, such as novelty of the open field arena and the size of the open field arena [108, 112, 113]. Previous studies found increased locomotor activity in young adult (PND 60) male rats compared to pubertal (PND 42) male rats when animals were placed into a novel open field arena, but this effect was not observed in rats that were habituated to the open field arena prior to testing to decrease spontaneous exploratory behaviors [108]. Furthermore, the size of the open field arena has been shown to impact the ability to observe sex differences in locomotor behavior. Increased locomotion by female rats compared to male rats has been observed in large open field arenas (129 × 120 × 60 cm) [112]. However, other studies have shown no sex differences in locomotor activity when using smaller open field arenas (70 × 70 × 70 cm or 54.5 × 80 × 33 cm) [113, 114]. It should be noted that in our study the rats were placed into a novel open field arena (40.64 × 40.64 × 38.1 cm), which may be why we observed age-associated changes in distance traveled locomotor activity but not sex-associated changes.

### Perspectives and significance

The major findings of this study are that gestational hypoxia has age- and sex-dependent effects on subcortical brain maturation in offspring, such as the nigrostriatal pathway. During puberty, female offspring were impacted more than male offspring. In contrast, male offspring were impacted more than female offspring during young adulthood, indicating long-term adverse effects in male offspring compared to female offspring. Impairment of cortical and subcortical pathway maturation, such as the nigrostriatal pathway, may increase risk for neuropsychiatric disorders (e.g., mood disorders, cognitive dysfunction, brain connectivity dysfunction). However, additional studies are needed to confirm this relationship between late gestational hypoxia and human neuropsychiatric disorders. It is noteworthy that CIH exposure during the last five days of gestation had long-term adverse effects on the offspring, while it did not affect the placentation process which occurs at the beginning of pregnancy, and had minimal impact on gestational outcomes. These novel findings demonstrate that gestational stressors can result in long-term adverse effects in offspring even in the absence of detectable pregnancy or neonatal complications, especially if they occur during critical embryological developmental periods.

## Supplementary Information

**Additional file 1: Table S1.** Summary of offspring total and categorized ultrasonic vocalizations. **Table S2. **Two-Way ANOVA results for call types and latency to first call.

## Data Availability

The datasets used and/or analyzed during the current study are available from the corresponding author upon reasonable request.
