# Supplementary material for: Gestational hypoxia in late pregnancy differentially programs subcortical brain maturation in male and female rat offspring
Source: Biol Sex Differ. 2022 Sep 30;13:54. doi: 10.1186/s13293-022-00463-x (PMC9524087; doi:10.1186/s13293-022-00463-x)
Supplement: Supplementary file 1 — Additional file 1: Table S1. Summary of offspring total and categorized ultrasonic vocalizations. Table S2. Two-Way ANOVA results for call types and latency to first call. [file 13293_2022_463_MOESM1_ESM.docx]

**Supplemental Table 1. Summary of offspring total and categorized ultrasonic vocalizations.**

| **Age Group** | ***In utero* Exposure** | **Sex** | **Total Calls** | **Chirp Calls** | **Simple Calls** | **Harmonic Calls** | **Other Calls** |
| --- | --- | --- | --- | --- | --- | --- | --- |
| Pubertal | Normoxia | Male | 20 | 5 | 4 | 10 | 1 |
|  |  |  | 6 | 3 | 2 | 1 | 0 |
|  |  |  | 0 | N/A | | | |
|  |  |  | 10 | 4 | 0 | 6 | 0 |
|  |  |  | 27 | 7 | 3 | 17 | 0 |
|  |  |  | 8 | 3 | 3 | 2 | 0 |
|  |  |  | 4 | 1 | 0 | 3 | 0 |
|  |  |  | 9 | 6 | 0 | 3 | 0 |
|  |  |  | 19 | 13 | 4 | 2 | 0 |
|  |  |  | 0 | N/A | | | |
|  |  |  | 2 | 2 | 0 | 0 | 0 |
|  |  |  | 5 | 3 | 1 | 1 | 0 |
|  |  |  | 2 | 0 | 2 | 0 | 0 |
|  |  |  | 7 | 3 | 3 | 1 | 0 |
|  |  |  | 24 | 10 | 3 | 11 | 0 |
|  |  |  | 1 | 0 | 0 | 1 | 0 |
|  |  | Female | 11 | 2 | 3 | 5 | 1 |
|  |  |  | 12 | 8 | 1 | 3 | 0 |
|  |  |  | 5 | 3 | 2 | 0 | 0 |
|  |  |  | 28 | 16 | 10 | 2 | 0 |
|  |  |  | 23 | 10 | 8 | 1 | 4 |
|  |  |  | 0 | N/A | | | |
|  |  |  | 0 | N/A | | | |
|  |  |  | 0 | N/A | | | |
|  |  |  | 1 | 1 | 0 | 0 | 0 |
|  |  |  | 8 | 7 | 1 | 0 | 0 |
|  |  |  | 8 | 4 | 3 | 1 | 0 |
|  |  |  | 61 | 8 | 29 | 10 | 14 |
|  |  |  | 57 | 25 | 13 | 17 | 2 |
|  |  |  | 37 | 23 | 4 | 10 | 0 |
|  | CIH | Male | 1 | 1 | 0 | 0 | 0 |
|  |  |  | 1 | 1 | 0 | 0 | 0 |
|  |  |  | 0 | N/A | | | |
|  |  |  | 0 | N/A | | | |
|  |  |  | 2 | 2 | 0 | 0 | 0 |
|  |  |  | 0 | N/A | | | |
|  |  |  | 8 | 4 | 3 | 1 | 0 |
|  |  |  | 6 | 6 | 0 | 0 | 0 |
|  |  |  | 3 | 2 | 0 | 1 | 0 |
|  |  |  | 2 | 2 | 0 | 0 | 0 |
|  |  |  | 65* | 13 | 38 | 3 | 11 |
|  |  |  | 24* | 14 | 6 | 4 | 0 |
|  |  |  | 0 | N/A | | | |
|  |  |  | 6 | 3 | 0 | 3 | 0 |
|  |  |  | 3 | 3 | 0 | 0 | 0 |
|  |  |  | 0 | N/A | | | |
|  |  |  | 3 | 2 | 1 | 0 | 0 |
|  |  |  | 13 | 8 | 2 | 3 | 0 |
|  |  |  | 2 | 0 | 0 | 2 | 0 |
|  |  |  | 21* | 8 | 3 | 10 | 0 |
|  |  | Female | 1 | 0 | 0 | 1 | 0 |
|  |  |  | 4 | 3 | 0 | 0 | 1 |
|  |  |  | 0 | N/A | | | |
|  |  |  | 6 | 2 | 4 | 0 | 0 |
|  |  |  | 4 | 3 | 1 | 0 | 0 |
|  |  |  | 2 | 1 | 1 | 0 | 0 |
|  |  |  | 2 | 1 | 1 | 0 | 0 |
|  |  |  | 4 | 2 | 0 | 2 | 0 |
|  |  |  | 9 | 4 | 1 | 4 | 0 |
|  |  |  | 0 | N/A | | | |
|  |  |  | 4 | 2 | 1 | 1 | 0 |
|  |  |  | 2 | 1 | 1 | 0 | 0 |
|  |  |  | 12 | 5 | 1 | 6 | 0 |
| Young Adult | Normoxia | Male | 10 | 7 | 1 | 2 | 0 |
|  |  |  | 0 | N/A | | | |
|  |  |  | 0 | N/A | | | |
|  |  |  | 8 | 5 | 3 | 0 | 0 |
|  |  |  | 1 | 1 | 0 | 0 | 0 |
|  |  |  | 0 | N/A | | | |
|  |  |  | 0 | N/A | | | |
|  |  |  | 9 | 2 | 4 | 3 | 0 |
|  |  |  | 4 | 4 | 0 | 0 | 0 |
|  |  |  | 0 | N/A | | | |
|  |  |  | 0 | N/A | | | |
|  |  |  | 8 | 5 | 1 | 2 | 0 |
|  |  |  | 0 | N/A | | | |
|  |  |  | 70* | 6 | 30 | 15 | 20 |
|  |  |  | 157* | 14 | 71 | 1 | 71 |
|  |  |  | 12 | 2 | 6 | 0 | 4 |
|  |  |  | 0 | N/A | | | |
|  |  |  | 23 | 4 | 4 | 15 | 0 |
|  |  |  | 4 | 0 | 1 | 3 | 0 |
|  |  | Female | 25* | 4 | 9 | 4 | 8 |
|  |  |  | 86* | 3 | 37 | 7 | 39 |
|  |  |  | 1 | 1 |  |  | 0 |
|  |  |  | 0 | N/A | | | |
|  |  |  | 0 | N/A | | | |
|  |  |  | 0 | N/A | | | |
|  |  |  | 3 | 2 |  | 1 | 0 |
|  |  |  | 1 | 0 | 1 | 0 | 0 |
|  |  |  | 5 | 2 | 0 | 2 | 1 |
|  |  |  | 6 | 1 | 3 | 0 | 2 |
|  |  |  | 63* | 8 | 30 | 8 | 17 |
|  |  |  | 38* | 13 | 20 | 1 | 4 |
|  |  |  | 7 | 6 |  | 1 | 0 |
|  |  |  | 6 | 4 | 9 | 2 | 0 |
|  | CIH | Male | 0 | N/A | | | |
|  |  |  | 10* | 2 | 0 | 8 | 0 |
|  |  |  | 4 | 2 | 1 | 0 | 1 |
|  |  |  | 1 | 1 | 0 | 0 | 0 |
|  |  |  | 1 | 0 | 0 | 1 | 0 |
|  |  |  | 6* | 5 | 0 | 1 | 0 |
|  |  |  | 1 | 1 | 0 | 0 | 0 |
|  |  |  | 0 | N/A | | | |
|  |  |  | 1 | 0 | 0 | 1 | 0 |
|  |  |  | 56* | 20 | 27 | 8 | 1 |
|  |  |  | 0 | N/A | | | |
|  |  |  | 2 | 0 | 1 | 0 | 1 |
|  |  |  | 2 | 1 | 1 | 0 | 0 |
|  |  |  | 2 | 2 | 0 | 0 | 0 |
|  |  |  | 0 | N/A | | | |
|  |  | Female | 0 | N/A | | | |
|  |  |  | 98* | 20 | 41 | 16 | 21 |
|  |  |  | 32* | 10 | 10 | 6 | 6 |
|  |  |  | 6 | 1 | 1 | 4 | 0 |
|  |  |  | 3 | 2 | 0 | 1 | 0 |
|  |  |  | 1 | 0 | 0 | 1 | 0 |
|  |  |  | 209* | 19 | 109 | 12 | 69 |
|  |  |  | 3 | 1 | 0 | 1 | 1 |
|  |  |  | 6 | 1 | 4 | 1 | 0 |
|  |  |  | 1 | 1 | 0 | 0 | 0 |
|  |  |  | 4 | 2 | 0 | 2 | 0 |
|  |  |  | 7 | 3 | 1 | 3 | 0 |

*, determined to be an outlier and removed from further statistical analysis. CIH = chronic intermittent hypoxia.

| **Call Type** | **Measure** | **Group** | **CIH** | | **Sex** | | **Interaction** | |
| --- | --- | --- | --- | --- | --- | --- | --- | --- |
|  |  |  | **F** | **P-value** | **F** | **P-value** | **F** | **P-value** |
| Total | Frequency | Puberty | 10.740 | ***0.002**** | 1.750 | 0.191 | 0.248 | 0.621 |
|  |  |  |  |  |  |  |  |  |
|  |  | Young Adult | 0.181 | 0.672 | 0.927 | 0.341 | 1.713 | 0.197 |
|  |  |  |  |  |  |  |  |  |
| N/A | Latency to First Call | Puberty | 3.018 | 0.089 | 2.800 | 0.101 | 0.260 | 0.612 |
|  |  | Young Adult | 0.149 | 0.702 | 1.938 | 0.172 | 6.155 | ***0.018**** |
| Chirp | Frequency | Puberty | 7.218 | ***0.010**** | 0.123 | 0.727 | 5.719 | ***0.021**** |
|  |  | Young Adult | 7.510 | ***0.010**** | 0.031 | 0.862 | 0.715 | 0.404 |
|  | Duration | Puberty | 4.912 | ***0.032**** | 1.324 | 0.256 | 2.696 | 0.108 |
|  |  | Young Adult | 0.273 | 0.605 | 2.362 | 0.134 | 0.712 | 0.405 |
|  | Intensity | Puberty | 0.471 | 0.496 | 0.902 | 0.348 | 0.205 | 0.653 |
|  |  | Young Adult | 0.171 | 0.682 | 0.004 | 0.950 | 0.959 | 0.334 |
|  | Bandwidth | Puberty | 0.015 | 0.903 | 1.164 | 0.287 | <0.0001 | 0.996 |
|  |  | Young Adult | 0.160 | 0.692 | 9.254 | ***0.005**** | 2.252 | 0.144 |
| Simple | Frequency | Puberty | 5.383 | ***0.028**** | 0.325 | 0.573 | 5.941 | ***0.022**** |
|  |  | Young Adult | 0.011 | 0.914 | 6.042 | ***0.024**** | 0.225 | 0.641 |
|  | Duration | Puberty | 0.289 | 0.596 | 1.839 | 0.186 | 0.703 | 0.409 |
|  |  | Young Adult | 0.510 | 0.483 | 3.864 | 0.063 | 2.153 | 0.157 |
|  | Intensity | Puberty | 0.029 | 0.865 | 0.591 | 0.448 | 0.211 | 0.650 |
|  |  | Young Adult | 6.111 | ***0.022**** | 0.862 | 0.364 | 10.010 | ***0.005**** |
|  | Bandwidth | Puberty | 1.096 | 0.304 | 0.210 | 0.650 | 0.874 | 0.358 |
|  |  | Young Adult | 0.583 | 0.454 | 5.135 | ***0.034**** | 0.005 | 0.943 |
| Harmonic | Frequency | Puberty | 1.114 | 0.300 | 0.265 | 0.611 | 0.952 | 0.338 |
|  |  | Young Adult | 0.319 | 0.578 | 0.570 | 0.457 | 0.353 | 0.558 |
|  | Duration | Puberty | 1.138 | 0.295 | 0.075 | 0.786 | 1.946 | 0.174 |
|  |  | Young Adult | 0.076 | 0.784 | 0.897 | 0.352 | 0.220 | 0.643 |
|  | Intensity | Puberty | 0.044 | 0.836 | 0.172 | 0.682 | 0.192 | 0.664 |
|  |  | Young Adult | 0.282 | 0.600 | 0.604 | 0.444 | 201.4 | 0.159 |
|  | Bandwidth | Puberty | 0.506 | 0.483 | 0.607 | 0.442 | 1.418 | 0.243 |
|  |  | Young Adult | 10.13 | ***0.004**** | 8.760 | ***0.007**** | 0.010 | 0.922 |

**Supplemental Table 2. Two-Way ANOVA results for call types and latency to first call.**

*****, p < 0.05. CIH = chronic intermittent hypoxia.
